# Bichloride of Mercury

**Published:** 1884-10-20

**Authors:** 


**Bichloride of Mercury.**—Dr. Curtiss says (in St. Louis Med. Rec.): I think this preparation of mercury comes the nearest to being a germitoxic that will kill germs without killing the patient of anything known at the present time. When the germs can be reached locally the drug is practically a scientific "cure" of the disease which may be caused by the germs. I think, also, that the old idea of the benefits of mercury in disease by defibrinating the blood, or modifying plastic exudation, is an error. Beyond a doubt most of the inflammatory diseases are germ diseases, and the plastic exudates are products of the action of germs upon the tissues—they are physiological products, just in the same sense that gall nuts and ergot are plastic exudates and pathological products; and mercury does not cure such things so much by its action upon the tissues as by its action upon the germs.

Relating to dosage, I give children from one-ninetieth to one-fortieth of a grain every four hours for scarlatina or diphtheria. I have never seen salivation, but I have never been obliged to continue the medicine more than three or four days, and, as I have said, there is no danger from cosmoline ointments, made for local use.

